# Ivory vertebra: imaging findings in different diagnoses*

**DOI:** 10.1590/0100-3984.2014.0103

**Published:** 2016

**Authors:** Richard Andreas Braun, Carlos Felipe do Rego Barros Milito, Suzan Menasce Goldman, Eloy de Ávila Fernandes

**Affiliations:** 1 Student Specializing in Radiology and Diagnostic Imaging in the Department of Diagnostic Imaging, Escola Paulista de Medicina da Universidade Federal de São Paulo/ (EPM-Unifesp), São Paulo, SP, Brazil.; 2 Student Specializing in Abdominal Radiology at the Instituto de Radiologia do Hospital das Clínicas da Faculdade de Medicina da Universidade de São Paulo (InRad/ HC-FMUSP), São Paulo, SP, Brazil.; 3 Tenured Affiliate Professor in the Department of Diagnostic Imaging, Escola Paulista de Medicina da Universidade Federal de São Paulo/ (EPM-Unifesp), São Paulo, SP, Brazil.; 4 Affiliate Professor in the Department of Diagnostic Imaging, Escola Paulista de Medicina da Universidade Federal de São Paulo/(EPM-Unifesp), São Paulo, SP, Brazil.

**Keywords:** Spine, Lymphoma, Neoplasm metastasis, Osteitis deformans, Osteomyelitis

## Abstract

Low back pain is often managed at all levels of healthcare. In general, diagnostic investigation begins with radiography of the lumbar spine. In addition to the most common findings, radiologists can identify increased density of a vertebral body, referred to as ivory vertebra. The objective of this study was to describe the main diseases that can present with this radiologic sign, such as Hodgkin lymphoma, Paget's disease, metastatic prostate cancer, breast cancer, and osteomyelitis. It is extremely important that radiologists be aware of this finding in order to inform the requesting physician of the possible etiologies, given that it can be the initial radiologic presentation for these diseases.

## Abstract (pt)

Pacientes com dor lombar são frequentemente atendidos em todos os níveis de saúde. O início da investigação diagnóstica, de modo geral, se dá com a realização das radiografias da coluna lombar. Além dos achados mais frequentes, os profissionais podem encontrar uma vértebra de densidade muito aumentada em comparação com as demais, chamada de vértebra em marfim. O objetivo deste trabalho é descrever as principais doenças que podem apresentar, entre suas características radiológicas, este tipo de vértebra, como linfoma de Hodgkin, doença de Paget, metástases de neoplasias prostáticas e de mama, além da osteomielite. Por ser, em alguns casos, a apresentação radiológica inicial dessas doenças, é de suma importância que o radiologista tenha conhecimento deste achado e oriente o profissional solicitante quanto às suas possíveis causas.

## INTRODUCTION

Using imaging methods to evaluate the musculoskeletal system is a current theme in the recent radiology literature from Brazil^(1-8)^. In general, radiography is the first step in imaging investigation of backbone diseases, followed by computed tomography (CT) or magnetic resonance imaging (MRI). The radiologist may find, during this primary investigation, vertebrae with increased density, with a "whiter" aspect than usual, similar to ivory, hence the designation ivory vertebra.

In order to interpret the images, it is important first to understand the normal anatomic characteristics and bone makeup of the vertebrae, and then the pathophysiology of the main diseases that may lead to ivory vertebra.

## BONE PHYSIOLOGY AND COMPOSITION OF THE VERTEBRAL BODIES

Bone tissue is a specialized connective tissue formed by a mineral phase, composed essentially of calcium phosphate crystals, in the form of hydroxyapatite, deposited in an organized collagenous matrix. This combination allows different bone types to be formed, from spongy bone to secondary bone tissue. These microscopic characteristics are in turn reflected in imaging aspects, such as cancellous bone and cortical, or compact, bone.

Despite their apparently inert aspect, bones are highly dynamic structures undergoing constant remodeling, where osteoclastic resorption is followed by the osteoblastic formation phase, promoting bone renewal.

Bone remodeling regulation may be affected by some diseases which promote an imbalance between bone deposition (density increase, as observed in the Hodgkin lymphoma ivory vertebra, blastic metastases, Paget's disease, and osteomyelitis) and resorption, particularly by osteoporosis.

## THE SPINE

**Figure 1:**
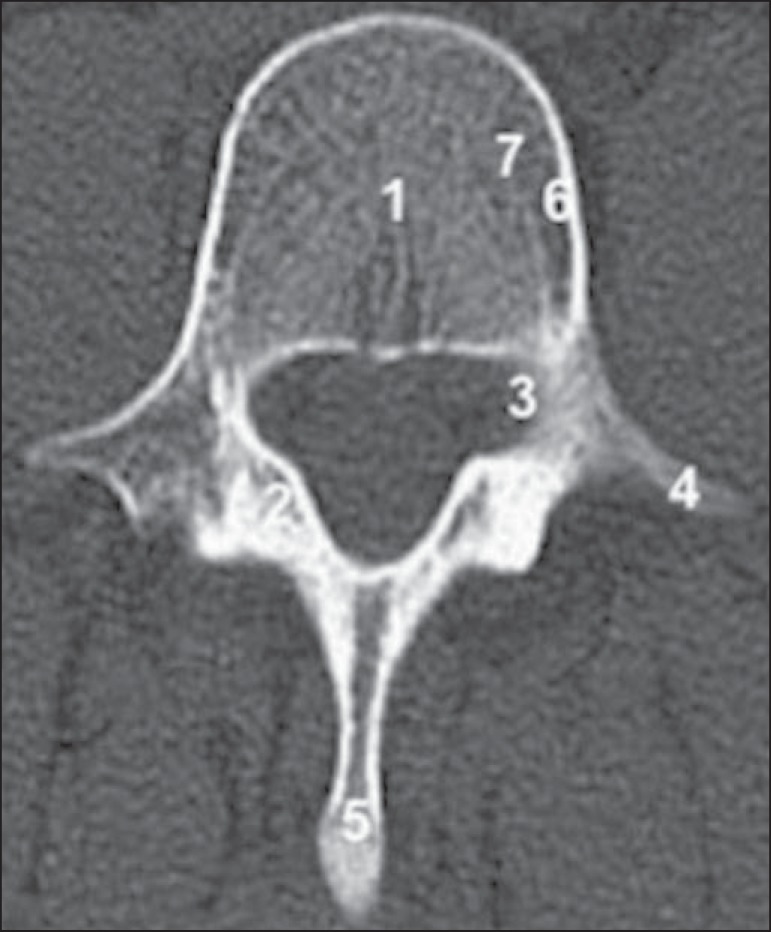
Computed tomography (cross section) of the backbone, L1 level, showing the vertebral elements, including the body (1), lamina (2), pedicles (3), transverse processes (4), and spinous process (5). The usual vertebral density of the cortical bone (6) and medullary bone (7) can also be seen.

The vertebral body is composed of vascularized trabecular bone (spongy bone). The trabecular interstices are filled with red and yellow bone marrow, covered by a thin external layer of compact bone, which appears with a dense aspect on CT scans and X-rays. Figure 1 shows the typical aspect of a normal-density vertebra.

Variations in vertebral bone density are typical of several diseases. The finding of an ivory vertebra means that one or more vertebrae has been affected by a density increase without any changes in the opacity or size of the adjacent intervertebral discs. Increased opacity of the vertebra may be diffuse and homogeneous, and most or the entire vertebral body is sclerotic, giving it a hyperdense appearance^(9)^.

Even though diffuse increases in vertebral density are generally referred to ivory vertebra, there are specific imaging characteristics that may increase diagnostic accuracy.

## DIFFERENTIAL DIAGNOSES

### Paget's disease

Paget's disease of bone, also known as osteitis deformans, is a hypermetabolic bone disease that attacks one (monostotic) or more (polyostotic) bones. It is marked by areas of increased osteoclastic bone resorption followed by disorganized osteoblastic bone repair. Consequently, the architecture of the affected bone tissue is disrupted, resulting in increased volume and greater bone fragility^(10)^.

It typically affects the skull, spine, pelvis, and long bones of the lower limbs^(10)^, although almost any part of the skeleton may be affected.

Bone involvement is frequently found in the elderly, where incidence varies from 2.3% to 9% in older patients. It generally appears after 55 years of age, with slight predominance in males^(11)^.

Most patients affected by Paget's disease of bone are asymptomatic, making it difficult to diagnose. In a study conducted by Collins, only 7 of the 24 cases (29%) were diagnosed while the patient was still alive^(11)^. When present, reported symptoms are pain, due to bone lesions or excessive bone growth, and deformities in the affected areas, such as osteoarthritis or nerve compression.

#### Characteristics that facilitate the diagnosis

**Figure 2:**
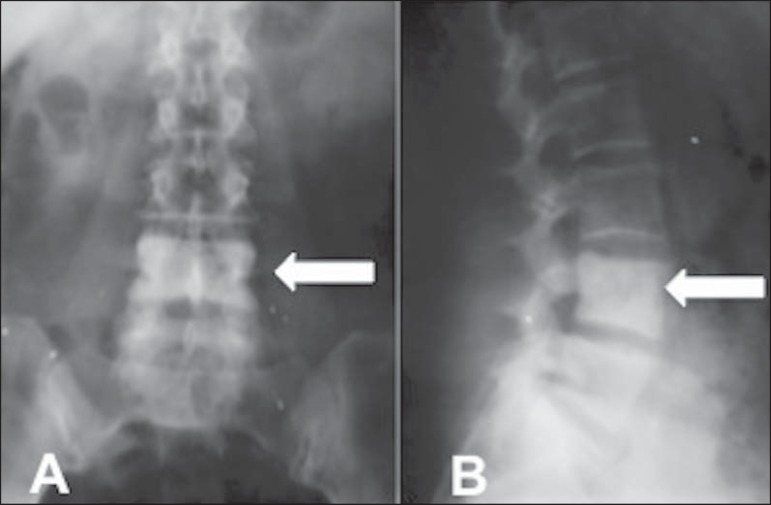
Asymptomatic 67-year-old patient. Anteroposterior (**A**) and profile (**B**) X-rays showing increased density of the L4 vertebral body (arrows).

In Paget's disease of bone, there is thickening and homogeneous hyperdensity of trabecular bone, as seen in Figure 2. An increase in anteroposterior and lateral diameters may also occur, resulting in an increase in vertebral size, in some cases leading to complications such as spinal stenosis and root compression. In its polyostotic form, the disease affects more than one site. A finding of an ivory vertebra that has no apparent cause and remains and unchanged over time is normally attributed to asymptomatic Paget's disease of bone.

### Lymphoma

Lymphomas are a heterogeneous group of primary neoplasms of the lymphoid tissue. Malignant lymphocytes normally accumulate on the lymph nodes, causing lymph node disease. Occasionally they may spread to the blood (leukemic phase) or infiltrate organs outside the lymphoid tissue. Lymphomas may be classified as Hodgkin disease, characterized by involvement of Reed-Sternberg cells, or non-Hodgkin lymphoma. Among all cases of lymphoma, 40-50% involve the skeletal system, generally from metastatic disease. This discovery, however, is frequently made only at autopsy, and not on X-rays^(12)^.

#### Imaging characteristics

Lymphomatous infiltration, originating from hematogenous dissemination or adjacent lymph node invasion, may have a marked effect on spongy bone. That involvement may lead to osteolysis, osteosclerosis, or a combination of the two. Lytic lesions are more common. Osteoblastic involvement is rare and generally secondary to Hodgkin lymphoma. In a study conducted by Granger et al.^(13)^, of 210 backbone lesions due to Hodgkin disease, only 13 had an ivory appearance.

**Figure 3:**
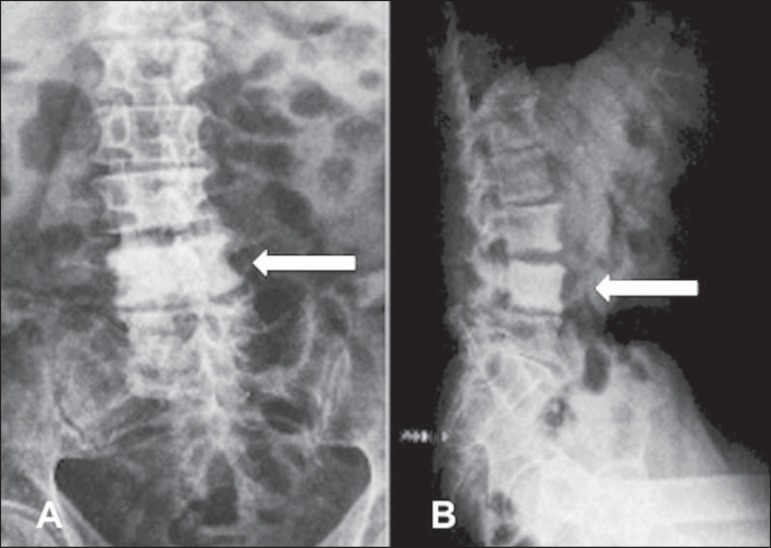
62-year-old patient with lymphoma and low back pain. Anteroposterior (**A**) and profile (**B**) X-rays showing a diffuse increase in bone density, with erosion on the left side of the L4 vertebral body (arrow).

Para-aortic lymph node involvement, which is typical of lymphoma, with the formation of paraspinal masses, may cause erosions in the anterior and lateral margins of the vertebral body cortex. Therefore, imaging findings such as an increase in trabecular size and density, which may result in an ivory vertebra, together with alterations in the adjacent soft tissue, help establish the diagnosis, as can be seen in Figure 3.

### Blastic metastases

Blastic metastatic lesions are most frequently the result of breast or prostate cancer, however, other metastatic tumors, including lymphoma, should be considered, together with other rare lesions, such as plasmacytoma, chordoma, and primary bone sarcoma^(9)^.

#### Imaging characteristics

In blastic metastases, osteoblasts are stimulated, resulting in irregular replacement of the spinal spongy tissue by amorphous and dense bone masses, which can converge. In imaging studies, these findings show up as an increase in vertebral density. Other characteristics that may increase the diagnostic specificity include the involvement of multiple spinal levels, blastic lesions in posterior spinal elements, and a known history of lung, kidney, or colon cancer, as well as, especially, prostate or breast cancer.

**Figure 4:**
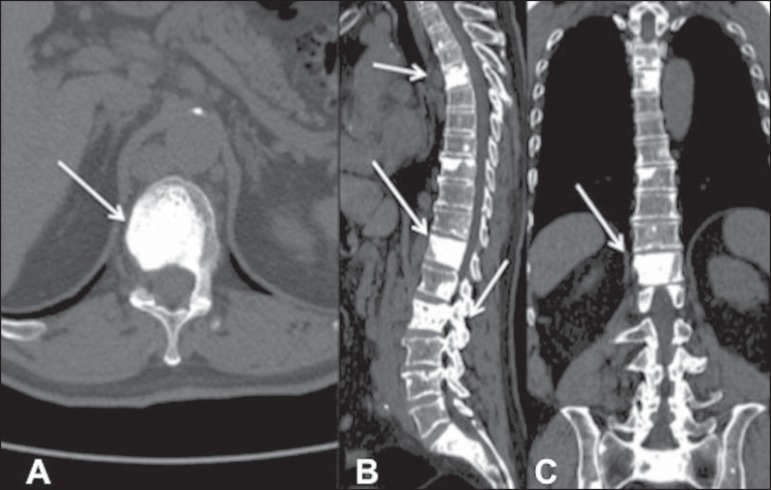
62-year-old patient with prostate cancer. computed tomography scans in the axial, sagittal, and coronal planes (**A**, **B**, and **C**, respectively), showing osteoblastic lesions with an increase in the density of the vertebral body and of the posterior vertebral elements (in **B**). Lesions are spread throughout the bone framework (arrows).

These characteristics can be seen in Figure 4, which shows a CT scan of a 62-year-old patient with primary prostate neoplasia. During staging, osteoblastic lesions were identified at various levels of the lumbar spine and hip.

**Figure 5:**
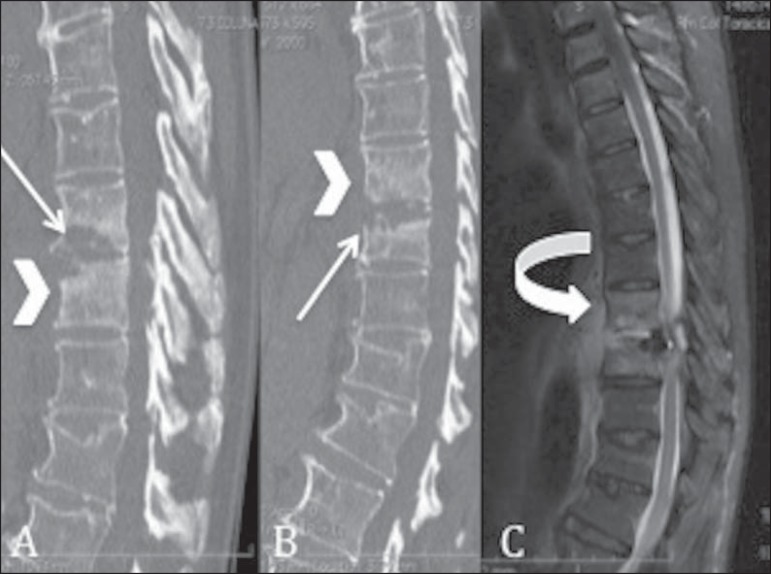
Computed tomography with sagittal reconstructions (**A,B**) and T2- weighted magnetic resonance imaging, in the sagittal plane, of the thoracolumbar transition (**C**). There is a density increase of the vertebral body (arrowheads) accompanied by bone erosion (arrows) at the margins of the intervertebral discs and collection (curved arrow) adjacent and anterior to the vertebral bodies (in **C**).

### Osteomyelitis

Osteomyelitis is a bone infection which leads to inflammatory destruction, followed by new bone formation. It is difficult to obtain viable information on the global incidence of vertebral osteomyelitis. In two studies, published in 1956 and 1991, the incidence of vertebral osteomyelitis was estimated at 1:250,000^(11)^ and 1:450,000^(14)^, respectively. Osteomyelitis may be classified as acute, subacute, or chronic.

#### Pathophysiology

After gaining access to the spine in one of three basic ways (hematogenous dissemination, direct inoculation, and contiguous propagation of infected adjacent soft tissues), pathogens cause an acute suppurative infection with inflammatory cells, followed by edema, vascular congestion, and thrombosis. In the initial acute disease, the blood supply to the bones is compromised because the infection extends to the surrounding tissue. When the blood supply to the bone marrow and the periosteum is compromised, areas of dead bone tissue may form. Due to the lack of blood supply, the dead bone is whiter than the surrounding bone, which indicates a loss of density. Although spongy bone is quickly resorbed and may be completely broken down in two to three weeks, the necrotic cortex may take two weeks to six months to appear^(15)^. With these characteristics, imaging findings for osteomyelitis are very specific, as can be seen on Figure 5. Therefore, sclerosis of a vertebra may occur during the healing phase and can be accompanied by erosions at the margins of the intervertebral disc, adjacent fluid collections, and predisposing factors or events, such as underlying diseases, hospitalization, invasive procedures, and intravenous drug use, all of which may inform the diagnosis.

## CONCLUSION

Ivory vertebra is sometimes the initial radiologic presentation for common diseases such as prostate carcinoma, lymphoma, and Paget's disease. It is therefore fundamental that radiologists be aware of its imaging characteristics, in order to better advise the requesting physician, and actively search for other lesions that might accompany it.

## References

[1] Maciel MJS, Tyng CJ, Barbosa PNVP (2014). Computed tomography-guided percutaneous biopsy of bone lesions:
rate of diagnostic success and complications. Radiol Bras.

[2] Terazaki CRT, Trippia CR, Trippia CH (2014). Synovial chondromatosis of the shoulder: imaging
findings. Radiol Bras.

[3] Arend CF (2014). The carpal boss: a review of different sonographic
findings. Radiol Bras.

[4] Arend CF (2014). Sonography of the iliotibial band: spectrum of
findings. Radiol Bras.

[5] Nakamura SA, Lorenzato MM, Engel EE (2013). Incidental enchondromas at knee magnetic resonance imaging:
intraobserver and interobserver agreement and prevalence of imaging
findings. Radiol Bras.

[6] Souza CG, Gasparetto EL, Marchiori E (2013). Pyogenic and tuberculous discitis: magnetic resonance imaging
findings for differential diagnosis. Radiol Bras.

[7] Machado BB, Lima CMAO, Junqueira FP (2013). Magnetic resonance imaging in intersection syndrome of the
forearm: iconographic essay. Radiol Bras.

[8] Alves MPT, Fonseca COP, Granjeiro JM (2013). Carpal tunnel syndrome: comparative study between sonographic and
surgical measurements of the median nerve in moderate and severe cases of
disease. Radiol Bras.

[9] Graham TS (2005). The ivory vertebra sign. Radiology.

[10] Altman RD, Bloch DA, Hochberg MC (2000). Prevalence of pelvic Paget's disease of bone in the United
States. J Bone Miner Res.

[11] Collins DH (1956). Paget's disease of bone; incidence and subclinical
forms. Lancet.

[12] Dennis JM (1961). The solitary dense vertebral body. Radiology.

[13] Granger W, Whitaker R (1967). Hodgkin's disease in bone, with special reference to periosteal
reaction. Br J Radiol.

[14] McHenry MC, Rehm SJ, Krajewski LP (1991). Vertebral osteomyelitis and aortic lesions: case report and
review. Rev Infect Dis.

[15] Shirtliff ME, Leid JG, Costerton JW, Calhoun JH, Mader JT (2003). The basic science of musculoskeletal infections. Musculoskeletal infections.

